# LAMP‐based molecular sexing in a gonochoric marine bivalve (*Macoma balthica rubra*) with divergent sex‐specific mitochondrial genomes

**DOI:** 10.1002/ece3.10320

**Published:** 2023-08-25

**Authors:** Sabrina Le Cam, Julie Brémaud, Tamás Malkócs, Eugénie Kreckelbergh, Vanessa Becquet, Emmanuel Dubillot, Pascale Garcia, Sophie Breton, Eric Pante

**Affiliations:** ^1^ Laboratoire Littoral Environnement et Sociétés (LIENSs) UMR 7266 CNRS – La Rochelle Université La Rochelle France; ^2^ Département de sciences biologiques Université de Montréal Montréal Québec Canada; ^3^ Laboratoire des Sciences de l'Environnement Marin (LEMAR) UMR 6539 CNRS‐UBO‐IRD‐Ifremer, Institut Universitaire Européen de la Mer Plouzané France

**Keywords:** DUI, heteroplasmy, loop‐mediated isothermal amplification, *Macoma balthica*, mitochondrial DNA, sexing

## Abstract

Taking advantage of the unique system of doubly uniparental inheritance (DUI) of mitochondria, we developed a reliable molecular method to sex individuals of the marine bivalve *Macoma balthica rubra*. In species with DUI (~100 known bivalves), both sexes transmit their mitochondria: males bear both a male‐ and female‐type mitogenome, while females bear only the female type. Male and female mitotypes are sufficiently divergent to reliably PCR‐amplify them specifically. Loop‐mediated isothermal amplification (LAMP) is a precise, economical and portable alternative to PCR for molecular sexing and we demonstrate its application in this context. We used 154 individuals sampled along the Atlantic coast of France and sexed microscopically by gonad examination to test for the congruence among gamete type, PCR sexing and LAMP sexing. We show an exact match among the sexing results from these three methods using the male and female mt‐*cox1* genes. DUI can be disrupted in inter‐specific hybrids, causing unexpected distribution of mitogenomes, such as homoplasmic males or heteroplasmic females. To our knowledge, DUI disruption at the intra‐specific scale has never been tested. We applied our sexing protocol to control for unexpected heteroplasmy caused by hybridization between divergent genetic lineages and found no evidence of disruption in the mode of mitochondrial inheritance in *M. balthica rubra*. We propose LAMP as a useful tool to accelerate eco‐evolutionary studies of DUI. It offers the opportunity to investigate the potential role of, previously unaccounted‐for, sex‐specific patterns such as sexual selection or sex‐specific dispersal bias in the evolution of free‐spawning benthic species.

## Graphical abstract

Our paper reports testing on the evolutionary stability of sex‐link mitochondrial heteroplasmy in males of the gonochoric bivalve *Macoma* *balthica* *rubra*, and the practical use of this stability to sex individuals molecularly, using loop‐mediated isothermal DNA amplification.

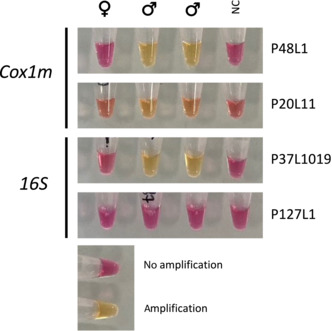

## 1 INTRODUCTION

Sex‐specific differences can play a major role in the evolution of gonochoric species. Interaction between sex‐differences and environmental changes can drive distinct evolutionary dynamics among sexes as a result of sex‐specific adaptation (Connallon et al., 2019). Yet they can be subtle and affect only early life history stages. Sex determination during adulthood can be extremely difficult, sometimes even for sexually dimorphic species (e.g. outside of fertility season). In ecological and population genetic studies, the adult sex ratio of a population is a fundamental parameter due, for example, to its effects on demography as biased sex ratios can lead to population collapse. In invertebrates, sex‐determination systems are highly diverse, (e.g. sex chromosomes, polygenic, environmental or cytoplasmic, reviewed Picard et al., 2021), and sex‐specific differences may be absent or subtle, so that sex ratios or sex‐specific patterns are rarely investigated in ecology or evolutionary biology. This is the case of bivalves, a group with an astonishing diversity of sexual systems, including one possible example of mitochondrial genes influencing sex determination pathways (Breton et al., 2018, 2022).

**FIGURE 1:**
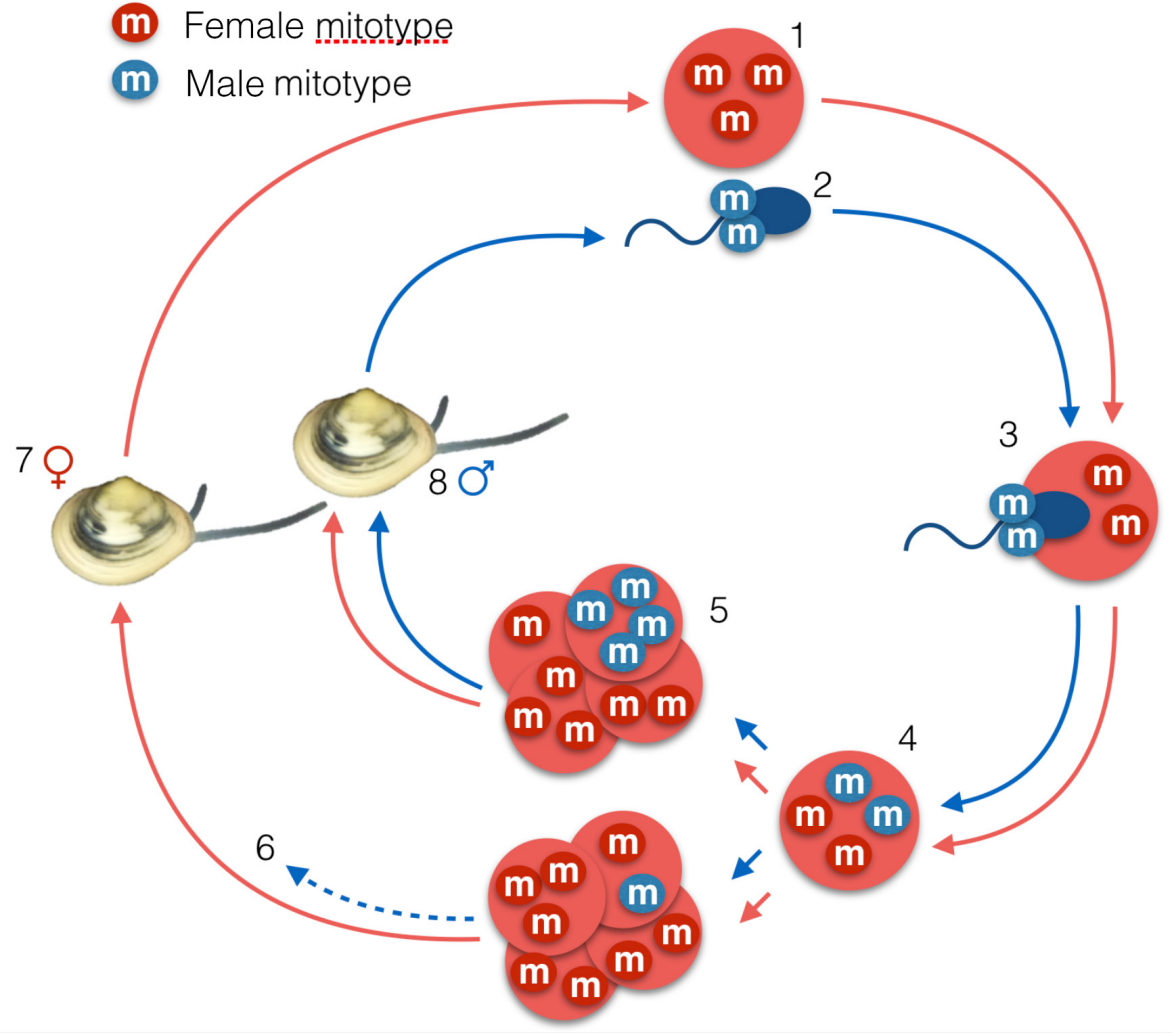
A model of doubly uniparental inheritance of mitochondria in bivalves. The ovocyte (1) and sperm (2) contain a single mitotype (homoplasmy). The sperm introduces the male mitotype into the ovocyte (3), and zygotes are therefore heteroplasmic (4). In early development, male mitotypes aggregate in the germ line of males (5), while they disperse (to the point of becoming undetectable) in females (6). At the adult stage, females (7) transmit the F mitotype with ovocytes while males (8) transmit the M mitotype with sperm (figure adapted from Breton et al. (2014)).

The only exception to the maternal inheritance of mitochondria in metazoans is found in bivalves. In the system of doubly uniparental mode of inheritance (DUI) of mitochondria, females transmit ‘female’ (F‐type) mitochondria that persist in oocytes and somatic tissues of both sexes, while males pass on ‘male’ (M‐type) mitochondria that persist in the male germ line (reviewed in Breton et al., 2007, 2014). This results in sex‐linked heteroplasmy, with females being mostly homoplasmic for the F‐type and males being heteroplasmic for the F‐ and M‐types (Figure 1). Among the ~11,000 nominal species of Bivalvia (Huber, 2010), DUI has been detected in over 100 (Gusman et al., 2016). One of the main characteristics of this system is the variable but generally high divergence between the F‐ and M‐type mitogenomes that may reach over 50% (Breton et al., 2007; Doucet‐Beaupré et al., 2010; Gusman et al., 2016). The DUI system offers a unique opportunity to design reliable molecular‐based sex determination tools for bivalve species (e.g. Dalpé et al., 2022; Kenchington et al., 2009).

Coevolution and coadaptation of mitochondrial and nuclear genes are required for efficient cellular energy production (i.e. oxidative phosphorylation OXPHOS) and mito‐nuclear genetic incompatibilities (MNIs) can lead to a desynchronization of this machinery (Burton et al., 2013; Burton & Barreto, 2012). Therefore, the DUI system offers tremendous potential for genetic incompatibilities to develop, particularly in inter‐populational hybrids, as the complex network of cytonuclear interactions can become disrupted due to the mixing of different gene pools (Saavedra et al., 1996). For example, interspecific hybridization (Zouros et al., 1994) can lead to heteroplasmic females and homoplasmic males for the F‐type in hybrids. In *Mytilus* mussels, this has been shown in experimental crosses (Wood et al., 2003; Zouros et al., 1994) and in natural populations (e.g. Rawson et al., 1996; Smietanka et al., 2013). Authors have postulated that mito‐nuclear genetic incompatibilities are involved in DUI disruption in hybrids and this could lead to reduced levels of introgression (e.g. Rawson et al., 1996).

**FIGURE 2:**
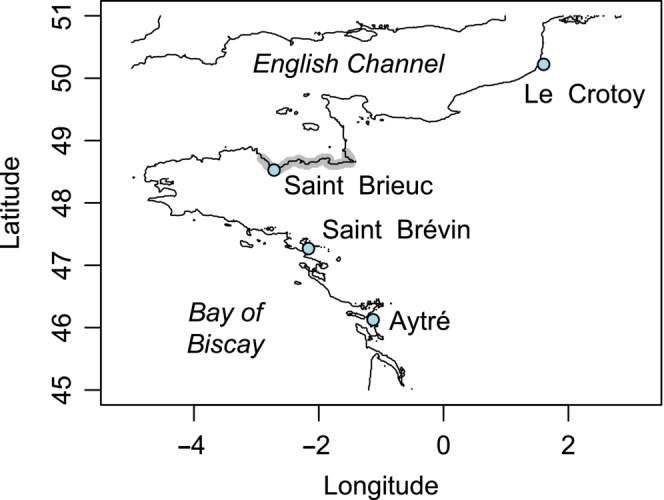
Sampling area. Study sites are depicted as blue dots. The location of the intra‐specific hybridization zone described in Becquet et al. (2012) and Le Cam et al. (2022) is represented as a thick gray line, and spans, based on current knowledge, from Saint‐Brieuc to Mont‐Saint‐Michel Bay.

*Macoma balthica* (Linnaeus, 1758) is a gonochoric bivalve characterized by DUI (Capt et al., 2020; Pante et al., 2017). Intra‐individual divergence between the M and the F mitotype can reach 54% at *nad6*, with an overall divergence level of 41% at the scale of the mitogenome (Capt et al., 2020). This species is broadly distributed along the coasts of the northern hemisphere, with populations in the eastern Atlantic spreading from the north of Russia (Hummel et al., 1997) to the Arcachon Basin (Hily, 2013; Le Cam et al., 2022). The northeastern Atlantic populations correspond to the sub‐species *M. balthica rubra* (Väinölä, 2003). The stretch of coast located between the Finistère and Cotentin Peninsulas harbours a transition zone between spatially separated genetic stocks (called thereafter ‘hybrid zone’ Figure 2; Becquet et al., 2012; their Figures 1 and 3). This area is characterized by strong population differentiation (*F*_ST_ = 0.48 at F‐type *cox1*, *F*_ST_ = 0.58 at M‐type *cox1*, Le Cam et al., 2022; nuclear coding SNP *F*_ST_ up to 1, Pante et al., 2012 and microsatellites *F*_ST_ = 0.021–0.035, Becquet et al., 2012) and geographically‐discordant genetic clines, with F‐type mtDNA being structured around the Finistère Peninsula, while the M‐type mtDNA is structured around the Cotentin Peninsula (Le Cam et al., 2022). To our knowledge, DUI disruption had not yet been investigated in intra‐specific hybrid zones.

**FIGURE 3:**
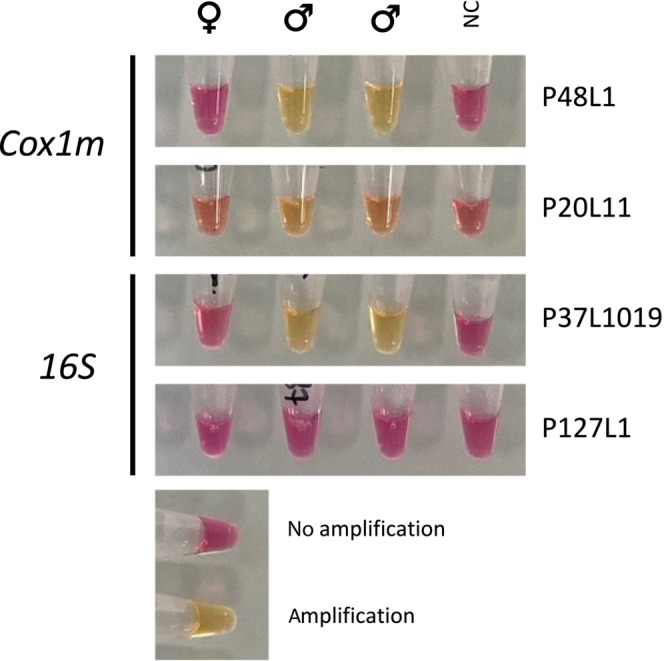
Loop‐mediated isothermal amplification (LAMP) of male *cox1* and 16S (*rrnL*) genes. Sample types: female (♀) and male (♂) gDNA templates and no‐gDNA negative control (NC). Names of primer sets (two per gene) are presented on the right. Yellow reactions correspond to successful amplifications of male‐type genes.

Since mito‐nuclear incompatibilities are candidate genetic barriers for the maintenance of the Finistère‐Cotentin hybrid zone in *M. balthica rubra*, we set to determine whether DUI showed any sign of disruption within this region. Under the model of DUI, female individuals are expected to be homoplasmic and thus only amplify for F‐type mtDNA, whereas male individuals are expected to be heteroplasmic and amplify for both M‐ and F‐types. The absence of disruption would have the practical advantage of allowing screening for male individuals within natural populations by way of molecular sexing. This technique, well established in wildlife sciences, consists in targeting the amplification of sex‐specific DNA to determine the sex of indistinguishable adults (e.g. when gonads are empty in species without sexual dimorphism) as well as any other life stages such as embryos (e.g. Mazzoleni et al., 2021) or larvae (e.g. Deng et al., 2020). This has been traditionally done with PCR, for over 30 years in some groups like birds (Morinha et al., 2012). In the context of DUI, molecular sexing may have multiple advantages, since it can be used to determine sexually‐immature individuals (from embryo to juvenile; e.g. Dalpé et al., 2022; Kenchington et al., 2009) or mature adults outside of their reproductive season and make use of legacy specimen collections for population studies. However, this technique remains labour‐intensive and requires gel electrophoresis.

In this study, we tested for the presence of disruption of the system of DUI of mitochondria inside and outside of the intra‐specific hybrid zone of the DUI bivalve *M. balthica rubra*. Based on our results, we went on to test the efficacy of molecular sexing by PCR and Loop‐mediated isothermal amplification (LAMP). The latter, developed in 2000 (Notomi et al., 2000) and more recently applied to wildlife molecular sexing, has several advantages over PCR sexing, including its simplicity, rapidity and previous successful applications in the field (Centeno‐Cuadros et al., 2018; Lee, 2017). So far, LAMP has been used in bivalves for pathogen detection (e.g. Durand et al., 2020), stock identification (Hashimoto et al., 2018) and alien species detection (Carvalho et al., 2021). Here, we show how LAMP sexing can be applied to detect the presence of male‐type mitochondria in tissues of a DUI species.

## 2 MATERIALS AND METHODS

### 2.1 Sampling

To detect the potential occurrence of DUI disruption in *Macoma balthica rubra*, individuals were sampled on both sides of the French hybrid zone that spans between the Finistère and the Cotentin peninsulas (Becquet et al., 2012; Le Cam et al., 2022). Northeast of this region, we sampled specimens in Somme Bay (Le Crotoy, abbreviated ‘CRO’). Southeast of the hybrid zone, we sampled in Aytré (‘AYT’, near the southern range limit of the species) and Saint‐Brévin‐les‐Pins (‘BRE’). Within the hybrid zone, we collected specimens in the Bay of Saint‐Brieuc (‘BRI’; Figure 2). At each sampling location, between 70 and 100 adult individuals from 11 to 23 mm (largest shell width) were collected, during the period of maximal sexual maturity (4th to 23rd of April 2018; Saunier, 2015). Individuals were then kept in aquaria until dissection with water temperature maintained at 10°C. They were fed with a multispecies microalgal mixture every other day; dead or dying individuals, if present, were removed daily.

Sex and gonadal maturation stage were determined by the identification of gametes with a dissecting microscope (×100 to ×400) following Saunier (2015). For each individual, the adductor muscle was carefully severed to separate the two valves and a sample of the mantle was taken. The gonad was then punctured with the tip of a scalpel and gametes were sampled with a P200 micropipet and shortened pipet tips. Each tissue sample (gonadic and somatic), along with the rest of the animal, was stored in separate 1.5 mL Eppendorf tubes, flash‐frozen in liquid nitrogen and then stored at −80°C until DNA extraction.

After microscopic sexing and tissue sampling, 18 DNA extractions were performed on mantle and gonads for nine males from three different sites crossing the Finistère/Cotentin hybrid zone: Aytré (3), Saint‐Brieuc (3) and Le Crotoy (3). Extractions from these two different tissue types were used to check that the male mitogenome could only be detected in the male gonad (details below). Next, 20 males and 20 females per geographical location (Aytré, Saint‐Brévin‐les‐Pins, Saint‐Brieuc, Le Crotoy; only 14 females could be reliably sexed at Le Crotoy) were selected to test for the amplification of the male mitochondrial genome from the gonads of both sexes (total: 154 individuals; 80 males and 74 females). A 3 mm^3^ piece of tissue was sampled from the gonad, which contains both somatic and gametic cells. Genomic DNA (gDNA) was purified with the Nucleospin Tissue Kit (Macherey Nagel), following the manufacturer's instructions. DNA quality and quantity were estimated using 1% agarose gel electrophoresis and quantification with NanoDrop2000. DNA extractions were performed with negative controls.

### 2.2 Design and validation of male‐ and female‐mtDNA PCR primers

Eight different primers (four forward and four reverse) were designed with Geneious v11.1.4 (Kearse et al., 2012) to specifically amplify either the 5′ portion of the male‐type (*cox1m*) or the female‐type (*cox1f*) *cox1* mitochondrial genes. We used female and male mitogenomes from Saunier et al. (2014) and Capt et al. (2020), as well as transcriptomic sequences from Pante et al. (2012) to design these primers. Seven primer pairs were tested on the 18 DNA extractions (nine somatic and nine gonad DNA samples) to confirm that *cox1m* primers are specific to male mt DNA and do not co‐amplify the female mtDNA partial gene (*cox1f*), and vice versa for *cox1f* primers. A single primer pair was finally chosen for each of *cox1m* and *cox1f*: cox1m14641F/cox1m15560R and cox1f5434F/cox1f6032R (Table 1). Polymerase chain reaction (PCR) amplifications of *cox1* gene fragments of the female (*cox1f*) and male (*cox1m*) mitochondrial DNA of the 154 DNA extractions were performed using the primers described above. PCR for *cox1* amplifications were realized in a 25 μL total volume, with 0.1 μL of Taq Polymerase 5 U/μL (TaKaRa Ex Taq® Kit MgCl_2_ Free Buffer; TaKaRa Ex Taq, TaKaRa Bio), 2.5 μL PCR Buffer 10×, 1.5 μL MgCl_2_ 25 mM, 1 μL dNTP 2.5 mM each, 0.6 μL each primer 10 μM and 17.7 Milli‐Q water. About 1 μL of extraction product with 1–5 ng of template DNA is used to amplify the *cox1m* and 5–20 ng to amplify the *cox1f*. PCR negative and positive controls were run; the DNA extraction blank was included in the PCR to test for trace DNA contaminants. SensoQuest thermal cyclers were used to perform the following PCR cycling profiles: for the *cox1f*, 2 min of initial denaturation at 94°C, then 30 cycles consisting of 45 s at 94°C followed by 30 s of annealing at 57°C and 40 s of elongation at 72°C and a 5 min final step of elongation at 72°C. For the *cox1m*, a different PCR cycling profile was used: 2 min at 94°C, then 30 cycles of 30 s at 94°C, 30 s at 60°C and 55 s at 72°C and a final elongation for 5 min at 72°C. PCR success (i.e. specificity and absence of amplicons for PCR and extraction negative controls), was tested on 1% agarose gels. PCR products were then sent to Eurofins GATC Biotech GmbH for Sanger sequencing to confirm that the *cox1m* and *cox1f* PCR products were homologous to recently published male and female mitogenomes. Sanger sequencing was performed in forward and reverse directions for all sequences. We used Geneious Prime v2019.1.2 (Kearse et al., 2012) to clean and contig forward and reverse traces. Finally, sequences were used to estimate the percentage of hybrid individuals (defined as individuals having discordant haplotypic signature) within and across the hybrid zone.

**TABLE 1:** List of PCR primers used for the amplification of the male *cox1m* and female *cox1f* mitochondrial genes.

Gene	Primer	Sequence (5′–3′)	Direction	*T*_M_ (°C)	Reference
cox1m	cox1m14641F	ATA GCT GGC CTG GTG TTT AGG	Forward	59.8	Le Cam et al. (2022)
	cox1m15560R	TTG GAC CCT TTC GAG CCA AG	Reverse	59.4	Le Cam et al. (2022)
cox1f	cox1f5434F	TTA GTG ACT TCA CAC GGT TTG C	Forward	58.4	Luttikhuisen et al. (2003)
	cox1f6032R	TGG GAA ATA ATC CCA AAC CCG	Reverse	57.9	Le Cam et al. (2022)

*Note*: *T*_M_ is the primer melting temperature.

### 2.3 Design and validation of male‐mtDNA LAMP primers for molecular sexing

Using the same comparative data from Capt et al. (2020), Pante et al. (2012) and Saunier et al. (2014), the LAMP primers were designed using the NEB® Primer Design Tools (neb.com/neb‐primer‐design‐tools, last accessed on 2022‐06‐1) for the *cox1* protein‐coding gene from the male mitogenome (*cox1m*). As a control, we performed the same analysis on the 16S (*rrnL*) ribosomal gene from the male mitogenome (16Sm). Four sets of LAMP primers were tested, 2 for *cox1m* and 2 for 16Sm. As for PCR primers, we checked that primers would be compatible with the range of genetic variation within *M. balthica rubra*. For that, primers were aligned to all the unique haplotypes of *cox1m* recorded in the distributional range of the focal species, that is from Aytré (France) to Lomma (Sweden; Le Cam et al., 2022; Figure S1). The region amplified by the LAMP primers ranged from the positions 535 to 747 of the entire *cox1* gene (Genbank accession number MH 285592.1). LAMP reactions were performed on the same set of individuals used for the DUI disruption study (154 individuals), using the WarmStart® colorimetric LAMP kit (E1700S; New England Biolabs), following the manufacturer's instructions (LAMP reaction was carried out in a total volume of 12.5 μL, including 1 μL of template containing 1–5 ng of gDNA, 6.25 μL of 2× LAMP buffer, 4 μL of Milli‐Q water and 1.25 μL of primer mix (16 μM of FIP, 16 μM of BIP, 4 μM of LB, 4 μM of LF, 2 μM of F3 and 2 μM of B3). Samples were incubated for 30 min at 65°C). Details and sequences of LAMP primers for the two best sets are presented in Table 2. Serial dilutions of male DNA from 10 to 0.01 ng/μL were performed to estimate the lower detection limit of sexing by LAMP.

**TABLE 2:** List of primers used for the detection of cox1m and 16Sm male‐type mitochondrial genes.

Gene	Set	Primer	*T*_M_ (°C)	Sequence (5′–3′)
cox1m	P48L1	F3	53.2	AGT ACA ATT ATC GGA ATG CG
		B3	56.4	CAA AAT AGG TGT ATA AAC AGA ACA G
		FIP	70.3	AAC CAC AAG TAA AAA AGC TGT GAT TAT CTG AAG GTA TGT CTA CCC A
		BIP	73.3	CTG TGC CTG TAT TAG CTG CTG CCA CAG GAT CAA AAA AAC ACG
		LF	57.9	GTT ACA AAC ATC CTC ATG CGG
		LB	57.3	GGC TTA CGA TGC TTC TAA CG
16Sm	P37L1019	F3	51.4	ATC TTG GTT GAG GCA TGT
		B3	56	TAT GCA GCC TAG TGA GCC
		FIP	71.2	GCA ACA TTG CGT ACA AAA AAG AGA AGT TAA GAA TGA GCT TGG GGT A
		BIP	71.1	TGC GAG AGT AAA CTA AGA TGG TGT ACT TAG ATG TAT TTC CTT TCC AAC
		LF	55.2	ACA GCC TAA CAT CCC GC
		LB	59.3	TAT GCT ATT CGA AAG GGC TGA TAG

*Note*: LAMP primer names are of six types: F3 (forward external primer), B3 (backward external primer), FIP (forward internal primer), BIP (backward internal primer), LF (loop forward primer), LB (loop backward primer) and *T*_M_ is the primer melting temperature.

### 2.4 LAMP applied to ‘Quick and Dirty’ DNA extractions from tissues

To test if the LAMP method could allow swift sex determination of *M. balthica*, it was tested on inexpensive and rapid DNA extractions (1 h preparation time). Ten individuals from Aytré and 10 from Le Crotoy were sampled and DNA was extracted using a modified method of the ‘Proteinase K method’ (Collard et al., 2007), in which tissue was added to Lysis Buffer T1 of the NucleoSpin Tissue kit (180 μL), mixed with 0.5 mg of Proteinase K, incubated at 56°C for 50 min. We used left‐over lysis buffer from the NucleoSpin Tissue kit used to purify total gDNA, yet any homemade lysis buffer can probably be used (as in Collard et al., 2007: TE buffer (10 mM Tris HCl, 1 mM EDTA pH 8.0)). Enzyme heat inactivation was carried out at 95°C for 10 min. After centrifugation at 12,000 *g* for 10 min, 100 μL of supernatant was retrieved. The 1/50 diluted gDNA extracts were used to amplify both *cox1f* and *cox1m* using standard PCR methods and *cox1m* using the LAMP method. Also, half of the remaining gDNA was treated further for DNA purification, following Macherey Nagel Nucleospin Tissue protocol.

## 3 RESULTS

### 3.1 PCR detection of male mitochondrial DNA and absence of DUI disruption

Among 18 tissue‐specific DNA extractions, *cox1m* PCR amplified for sperm but not for mantle tissue; *cox1f* amplified for somatic tissues of both sexes and oocytes. Among our 154 gonadal gDNA extractions, *cox1f* successfully amplified for 100% of male individuals (i.e. 80/80, Figure S2) and for 100% of female individuals (i.e. 74/74, Figure S3). *cox1m* amplified for 100% of males (Figure S4) and failed in all the females but one (Figure S5A,B). A faint *cox1m* PCR band could be seen for one female (Le Cro10) and it was confirmed by LAMP (see after). We re‐extracted the DNA from the gonad of this individual and confirmed the male ‘diagnostic’ suggested by the first extraction/amplification (Figure S5C). After going back to the field notes, we found that this outcome was the result of a book‐keeping error during sampling. Sequencing of PCR products confirmed the homology of these fragments with known male‐type and female‐type *cox1* haplotypes (Capt et al., 2020; Saunier et al., 2014), and yielded sequences of 676 and 479 bp for *cox1m* and *cox1f*, respectively. Sequences are further compared in a separate phylogeographic study (Le Cam et al., 2022).

### 3.2 LAMP molecular sexing

Among the four LAMP primer sets, two provided unambiguous results for molecular sex‐determination, one for 16S and one for *cox1* (P37L1019 and P48L1, respectively) (Figure 3). The *cox1* P48L1 primer set was used for further investigations. First, the 154 individuals used in the disruption study were tested to investigate the potential of the LAMP method in rapid molecular sexing. Some of the stock DNA were empty by the time of the experiment (two females and seven males) and 10 μL of RNAse‐free water was added to the tube in an attempt to test them but failed (see Figures S6 and S7). One hundred per cent of the 73 males presented a positive result (Figure S6). Seventy out of 72 females presented negative results (no colour change) as expected (Figure S7). The incongruent amplifications among females were specimens from Le Crotoy (Le Cro 10, confirmed male after re‐extraction, see above) and Saint‐Brévin‐les‐Pins (B19; DNA was re‐extracted). For B19, the DNA from the gonad was also re‐extracted and this time, the ‘female status’ was confirmed. There was congruence between microscopic, PCR and LAMP sexing was confirmed. This suggested that the previous positive LAMP signal for this individual was most likely the result of cross‐contamination between two samples during the original DNA extraction rather than a false positive.

### 3.3 LAMP applied to ‘quick and dirty’ DNA extraction

The DNA of 20 individuals (10 from Aytré and 10 from Le Crotoy) was successfully extracted using the quick and dirty (Q&D) method. Three samples were randomly chosen for quality control. DNA extractions were highly concentrated (from 1.2 to 1.6 μg/μL) but contained high levels of contaminants (A260/A280: 1.73–1.91; A260/A230: 0.28–0.31), as expected. DNA purification was carried out using the Macherey Nagel Tissue kit products and protocol, resulting in lower DNA concentration (13.7–30 ng/μL) but higher purity (A260/A280: 1.74–2.01; A260/A230: 1.27–1.97).

The LAMP method successfully worked on 1/50 dilutions of unpurified Q&D DNA extractions (24–32 ng/μL), whereas standard PCR did not. PCR was, therefore, carried out on undiluted purified DNA (13.7–30 ng/μL) and showed 100% congruence with the LAMP results (14 males and 6 females, Figure S8). Serial dilutions of genomic DNA suggested that LAMP can amplify *cox1m* down to a concentration of 0.1 ng/μL.

## 4 DISCUSSION

### 4.1 Prevalence of intra‐specific hybrids at the study sites

Based on the heteroplasmic state of males in DUI species, we were able to document the prevalence of intra‐specific hybrids in males at the study sites (Le Cam et al., 2022). At Le Crotoy (northern population), 86% of individuals belonged to the northern *cox1m* (m2/m3 clades) and *cox1f* haplogroups (b1a and b2/b3 clades; Le Cam et al., 2022). At Saint Brévin‐les‐Pins and Aytré, 65% and 73% of individuals belonged to the southern haplogroups (clade b1b at *cox1f* and m1 at *cox1m*; Le Cam et al., 2022). At Saint‐Brieuc, within the hybrid zone, all 30 males had discordant *cox1* haplotypes: all belonged to the southern *cox1m* haplogroup (‘m3’ sensus; Le Cam et al., 2022) but had a central/northern *cox1f* signature (16 individuals from the northern b2/b3 clades, 14 from the central b1a clade). However, a third of females had discordant *cox1* signatures: 11 individuals were from the northern b2/b3 clade, 10 from the central b1a clade and nine from the southern b1b clade. The biogeography of males based on the paternally‐inherited *cox1m* and maternally‐inherited *cox1f* gene markers is described in detail in Le Cam et al. (2022).

There was no evidence for the disruption of doubly uniparental inheritance of mtDNA in males or females within the studied intra‐specific hybrid zone, as no heteroplasmic females or homoplasmic males were detected. Replicated sampling in this region suggests temporal stability of the hybrid zone between 2003 (Becquet et al., 2012) and 2018 (Le Cam et al., 2022). A single type of somatic tissue (mantle) was considered, and it is possible that heteroplasmy in females may be restricted to other tissues, although this has not been reported in the literature to our knowledge. In addition, homoplasmy in males was never detected either. The genealogies of the *cox1m* and *cox1f* haplotypes, used for searching for disruption, are known (Le Cam et al., 2022), and it is, therefore, highly unlikely that lack of evidence of heteroplasmy in females may be due to primer design. We can therefore presume that inter‐lineage hybridization does not affect mitochondrial inheritance in this system. Opposed to this interpretation, it could be hypothesized that disruption of DUI (or sex‐determination mechanisms associated with DUI) is lethal in *M. balthica rubra* and leaves no trace in natural populations. We could also envision that disruption is rare enough not to be detected in a sample of 154 specimens. This could be further investigated with experimental crosses (e.g. Wood et al., 2003; Zouros et al., 1994). That said, DUI disruption through hybridization is not always occurring in *Mytilus* mussels: while hybrids between *M. trossulus* and *M. galloprovincialis* as well as *M. trossulus* and *M.edulis* show signs of disruption, *M. galloprovincialis* × *M.edulis* hybrids do not (Rawson et al., 1996). This difference was attributed to the lower genetic divergence of the *M. galloprovincialis*/*M.edulis* pair compared to *M. trossulus*. The divergence time between populations on either side of the Finistère/Cotentin peninsula is probably younger (0.11–2.6 Mya; Luttikhuisen et al., 2003) than for the *Mytilus* species pairs (TimeTree of Life: 1.880–2.474 Mya; Plazzi & Passamonti, 2010; Ren et al., 2010), supporting this hypothesis. Further, the genetic divergence between the northern, central and southern haplogroups is low for both *cox1m* and *cox1f*: along the 479 bp fragment consider, one to four mutational steps separate these clades, and a maximum of 25 and 26 mutational steps separate the most divergent haplotypes for *cox1f* and *cox1m*, respectively (Le Cam et al., 2022). A better understanding of the phenomenon of disruption may therefore emerge from the study of hybridization among more diverged *Macoma* lineages or in some other bivalve species.

Lack of disruption of DUI in *M. balthica rubra* means that males and females can be reliably sexed based on the presence/absence of male mitochondrial haplotypes in gonadal tissue, irrespective of the nature of the coupling between DUI and sex determination (which can be causative or associative; Kenchington et al., 2009; Zouros, 2013). There was a complete correspondence between sex as determined by microscopy, PCR amplification and LAMP. Each technique has its advantages. Microscopy is economical (virtually free aside from equipment), fast and reliable and allows the detection of other phenomena such as endocrine disfunction (e.g. in the form of ovotestis, or oocytes in the testes, in the DUI species *Scrobicularia plana*; Chesman & Langston, 2006), detection of rare hermaphroditic individuals (e.g. in the venerid clam *Megapitaria squalida*, Piñera et al., 2009), parasitism (Saunier, 2015) or neoplasia (Michnowska et al., 2022). However, during most of the year (May to March) specimens do not have differentiated gametes, so microscopic sexing is impossible. Despite its well‐known advantages, PCR is more expensive compared to LAMP, more time‐consuming (few hours) and may require a prerequisite step of performant DNA isolation and a subsequent electrophoresis. However, it is highly sensitive (in theory a single DNA molecular can elicit PCR amplification) and it allowed molecular sexing of hundreds of specimens of *M. balthica* from legacy collections (20+ year‐old specimens, S. Le Cam, V. Huet, E. Pante, unpublished data). LAMP is affordable, fast (5 min bench time and 30 min reaction for LAMP) and highly portable. It can be done in the field using a portable heat source (hot water bath, thermal cycler or heat block) and minimal DNA extraction (Centeno‐Cuadros et al., 2017). Moreover, LAMP relies on a set of six primers to function, compared to two for PCR. It is therefore theoretically more sensitive than PCR and less prone to false positives. For instance, Hamburger et al. (2013) found that LAMP detection of schistosome‐infected snails was 10 times more sensitive than PCR. We have found that LAMP also works with products of rapid and cheap extraction method (Q&D DNA extraction) where PCR is not working. Combination of Q&D extraction and LAMP allows large‐scale molecular sexing in a limited time (~2 h for 32 samples). Another important difference between PCR and LAMP is the nature of false negatives: while sexing PCR can use M‐ and F‐specific primers in tandem, hence significantly reducing the incidence of false negatives due to DNA quality or quantity, LAMP relies on the amplification of a single, small locus (212 bp‐long in our case). While a higher incidence of false negatives is a possibility, we found none in our dataset. In fact, the small size of the LAMP amplification region probably contributes to its sensitivity. Depending on the application (e.g. detect males to carry out further experiments vs estimate the population sex ratio), a false positive will not carry the same significance. For the latter, an additional set of LAMP primers designed on the F‐type *cox1* gene would be necessary to discard DNA extraction failure. In conclusion, LAMP can therefore be an interesting technique in the toolbox of DUI researchers, providing a sensitive method for the detection of male‐type mitochondria in all taxa and sexing for groups unaffected by DUI disruption. This method could open a whole new chapter in eco‐evolutionary studies in DUI species as it allows, in free spawning benthic invertebrates, to investigate sex‐specific patterns (dispersal, selection, hybridization) and their role in evolutionary processes.

## AUTHOR CONTRIBUTIONS

**Sabrina Le Cam:** Conceptualization (equal); methodology (lead); validation (lead); writing – original draft (equal); writing – review and editing (equal). **Julie Brémaud:** Methodology (equal); validation (equal); writing – original draft (supporting); writing – review and editing (supporting). **Tamás Malkócs:** Methodology (supporting); validation (supporting); writing – review and editing (supporting). **Eugénie Kreckelbergh:** Methodology (supporting); validation (supporting); writing – review and editing (supporting). **Vanessa Becquet:** Writing – review and editing (supporting). **Emmanuel Dubillot:** Resources (supporting). **Pascale Garcia:** Funding acquisition (equal); writing – review and editing (equal). **Sophie Breton:** Investigation (supporting); writing – original draft (supporting); writing – review and editing (supporting). **Eric Pante:** Conceptualization (equal); funding acquisition (lead); methodology (lead); project administration (lead); resources (equal); supervision (equal); validation (equal); writing – original draft (equal); writing – review and editing (equal).

## FUNDING INFORMATION

This work was funded by the ANR (DRIVE project, grant n. ANR‐18‐CE02‐0004‐01) and by the Contrat de Plan Etat‐Région (CPER/FEDER) ECONAT (RPC DYPOMAR).

## BENEFIT‐SHARING STATEMENT

A research collaboration was developed with scientists from different countries around the eco‐evolutionary studies of DUI species, Benefits from this research accrue from the sharing of our data.

## Supporting information


Figure S1

## Data Availability

All data are provided in the manuscript (molecular protocol, primer sequence). Photos of LAMP and PCR gel results are presented in Supporting Information.
