# Supplementary material for: LAMP‐based molecular sexing in a gonochoric marine bivalve (Macoma balthica rubra) with divergent sex‐specific mitochondrial genomes
Source: Ecol Evol. 2023 Aug 25;13(8):e10320. doi: 10.1002/ece3.10320 (PMC10450836; doi:10.1002/ece3.10320)

Supplementary Material : **LAMP-based molecular sexing in a gonochoric marine bivalve *(Macoma balthica rubra)* with divergent sex-specific mitochondrial genomes**

Fig S1: Alignment of the LAMP primers (P48_L1) and the unique haplotypes of *cox1m* used to design them.


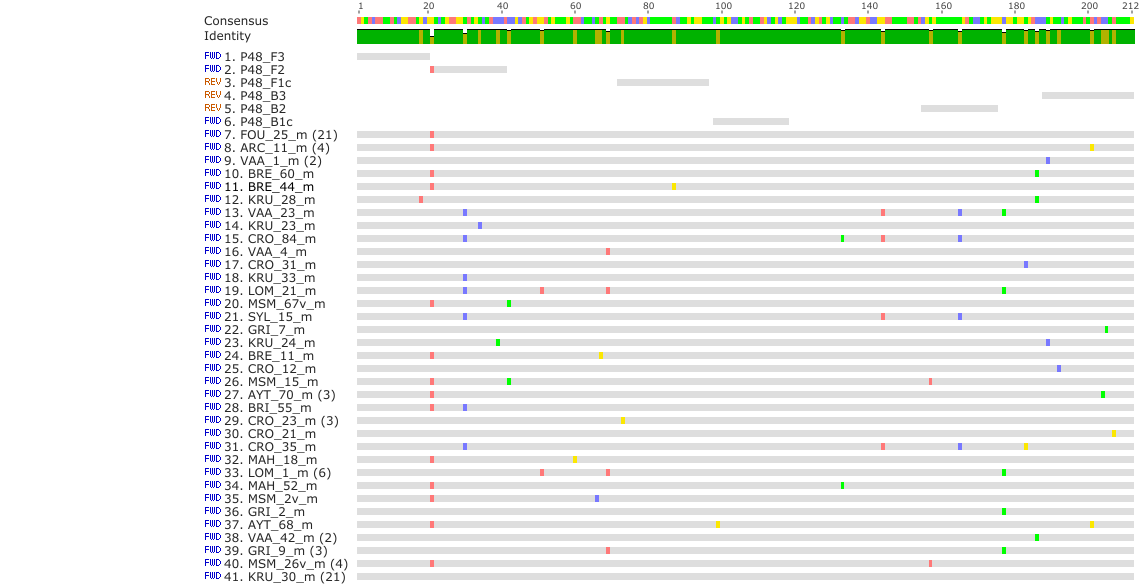


Figure S2: PCR amplification of *cox1f* on 74 females (5µl of PCR product run on a 1.5% agarose gel for c.a. 45 min at 90V)

Figure S3: PCR amplification of cox1f on 80 males (5µl of PCR product run on a 1.5% agarose gel for c.a. 45 min at 90V)


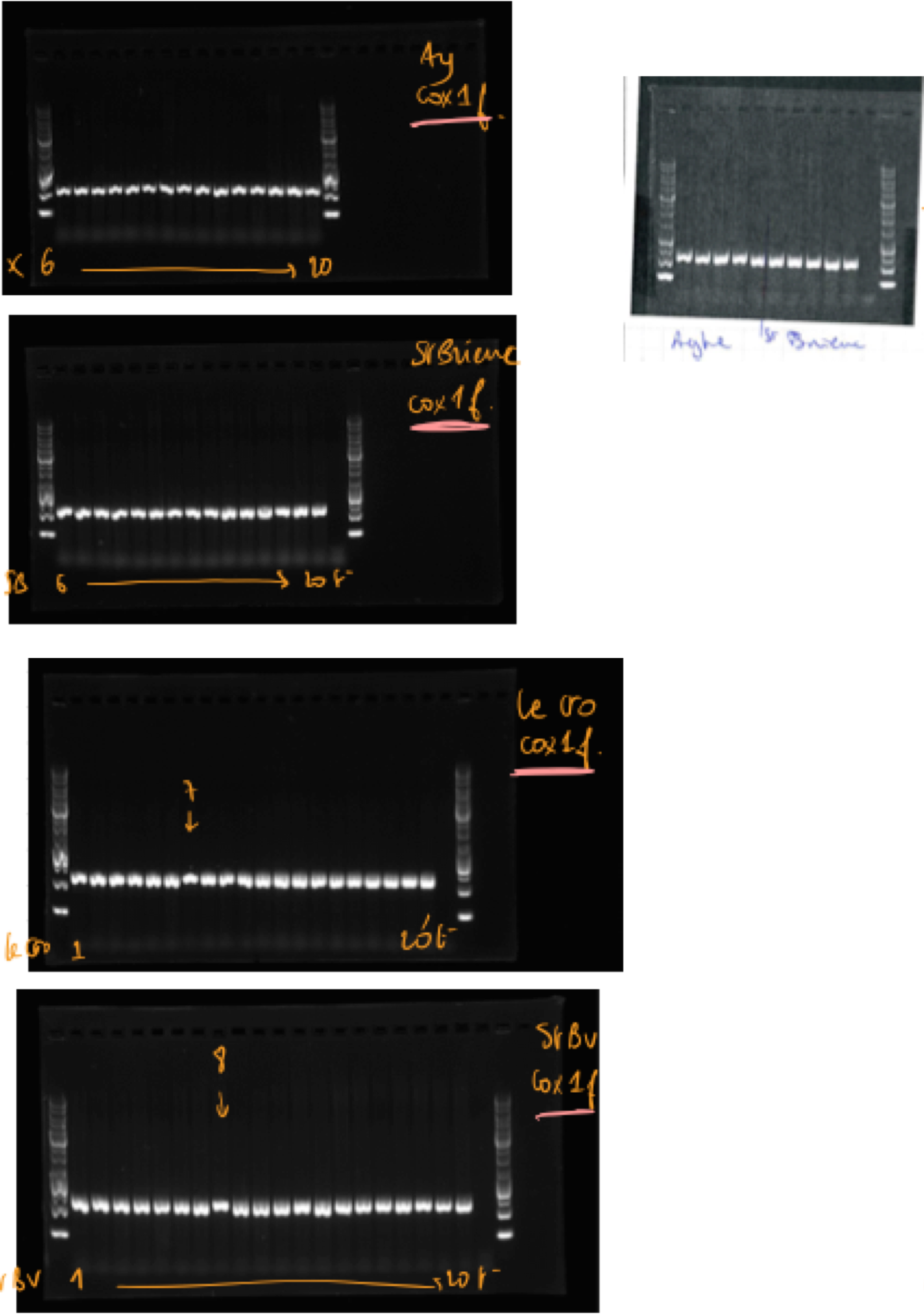


Figure S4: PCR amplification of *cox1m* on 80 males (5µl of PCR product run on a 1.5% agarose gel for c.a. 45 min at 90V)


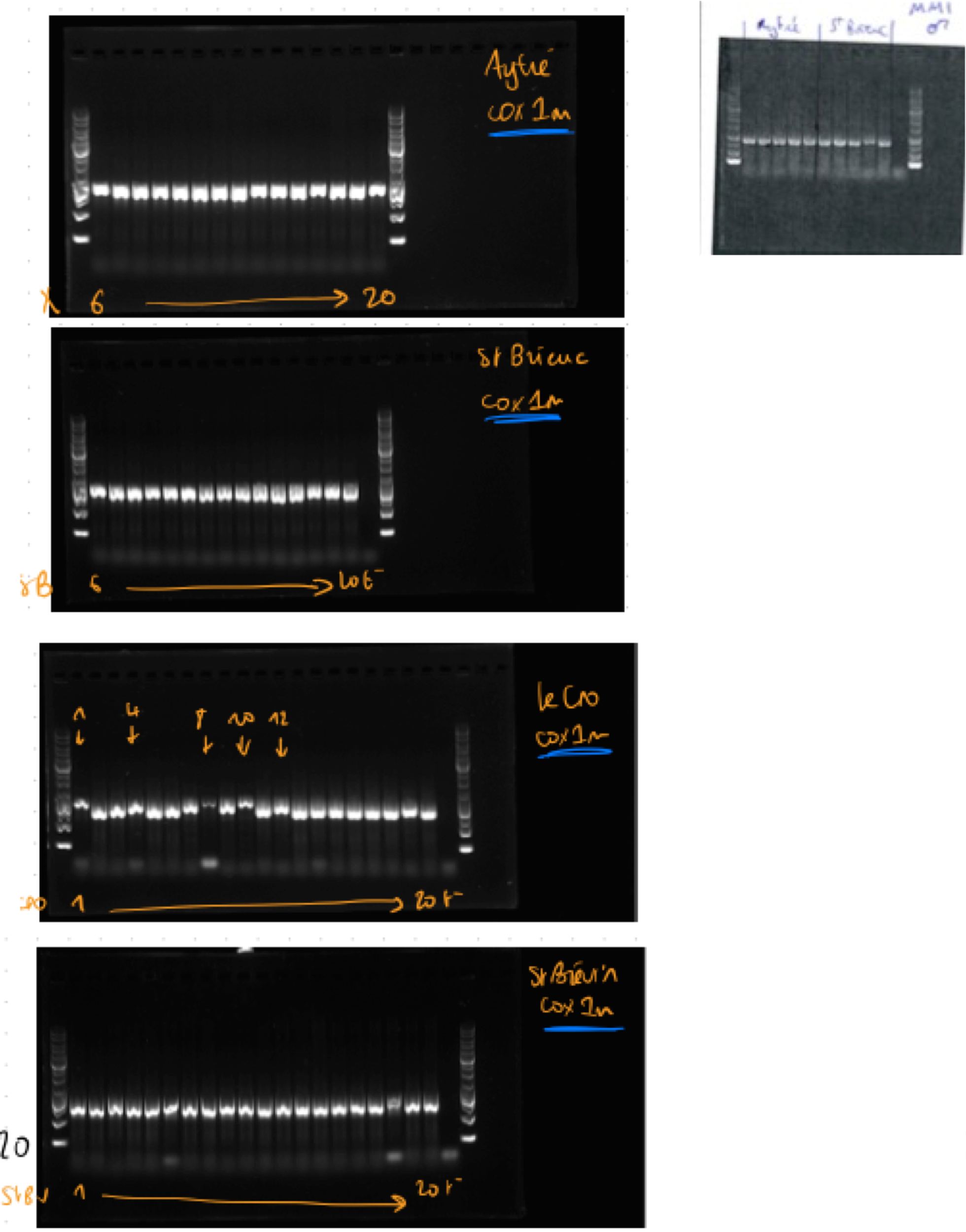


Figure S5: PCR amplification of *cox1m* on 74 females (5µl of PCR product run on a 1.5% agarose gel for c.a. 45 min at 90V)


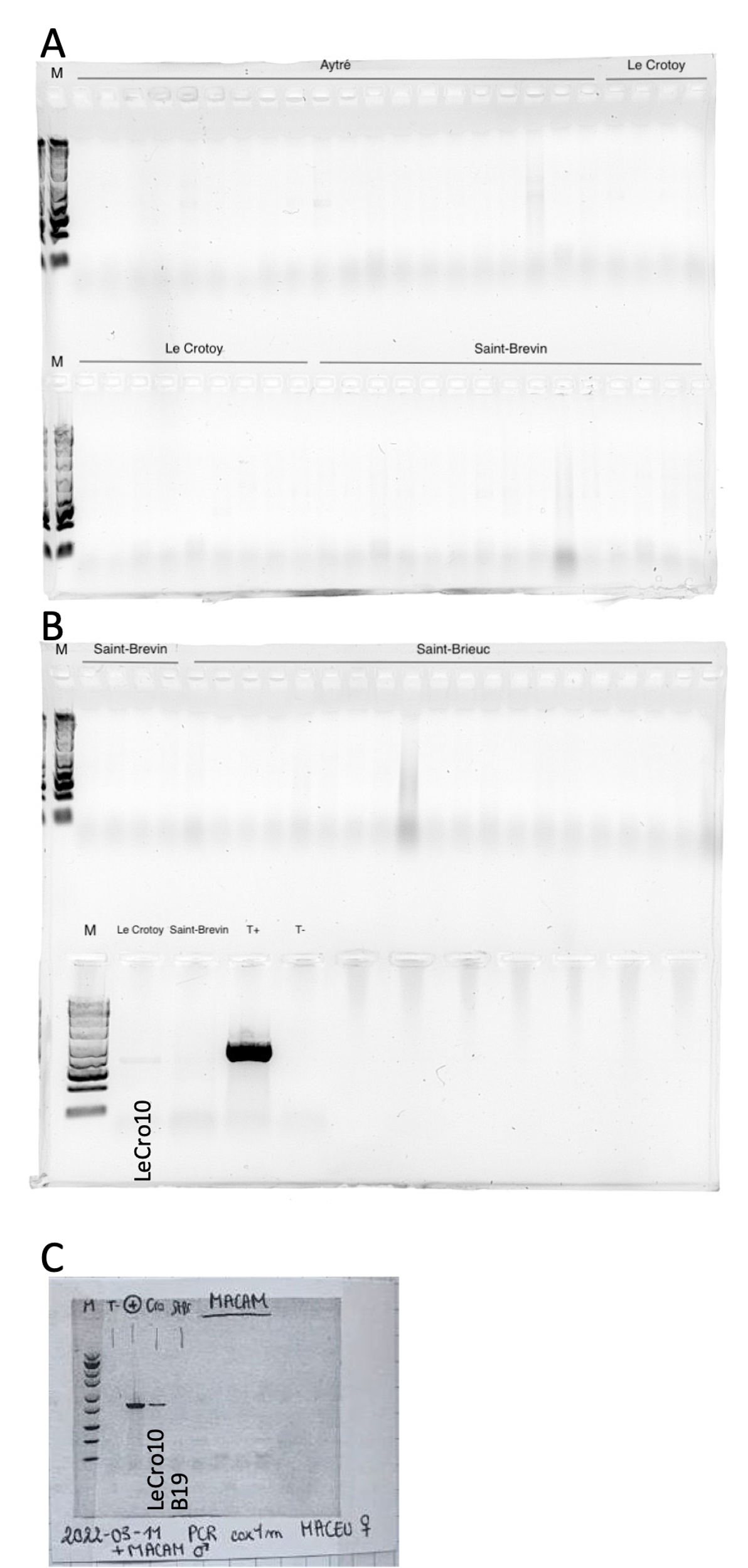


Figure S6: LAMP amplification results of 80 males


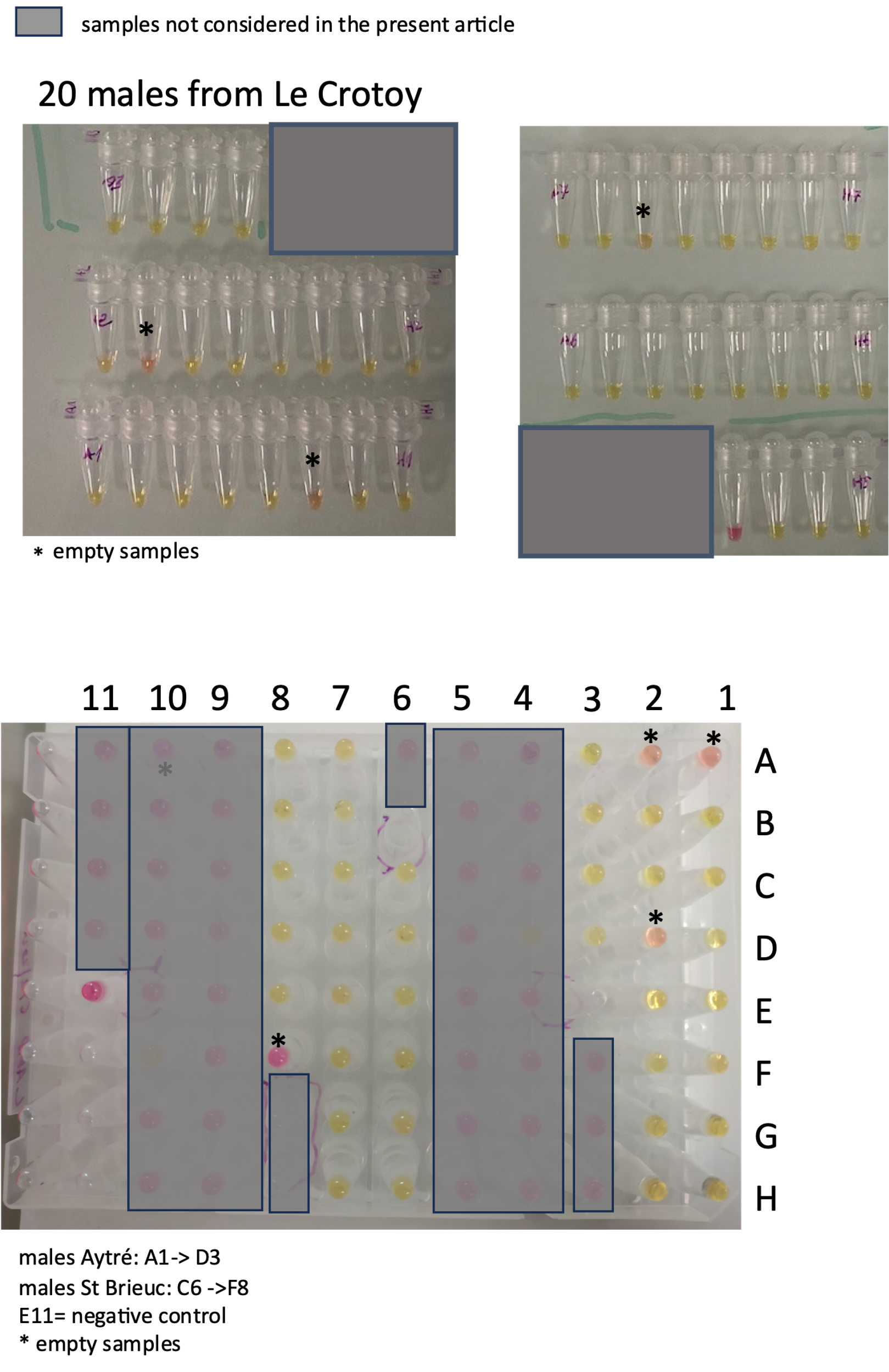


Figure S7: LAMP amplification results of 74 females


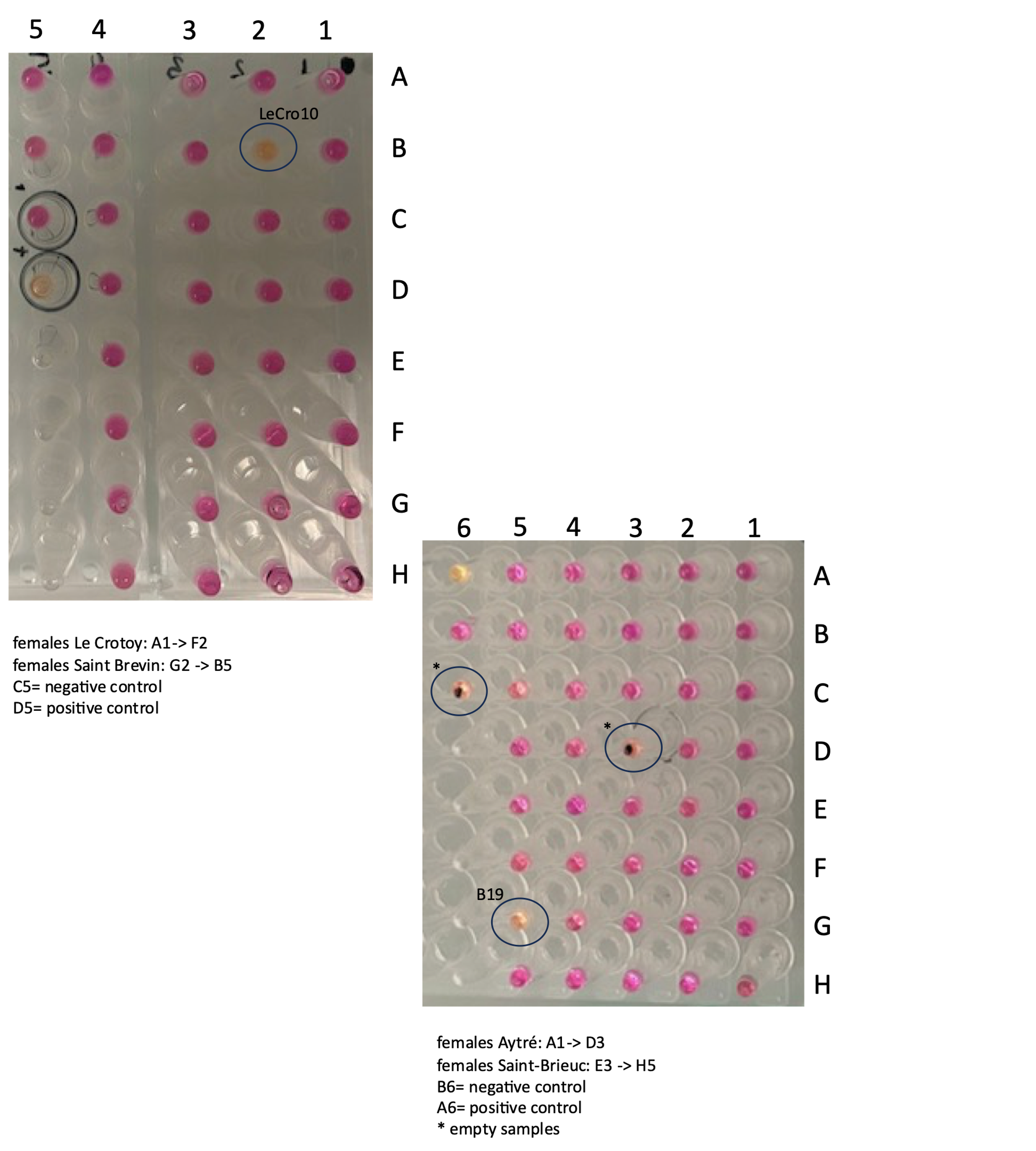


Figure S8: Molecular sexing following ”quick and dirty” DNA extraction


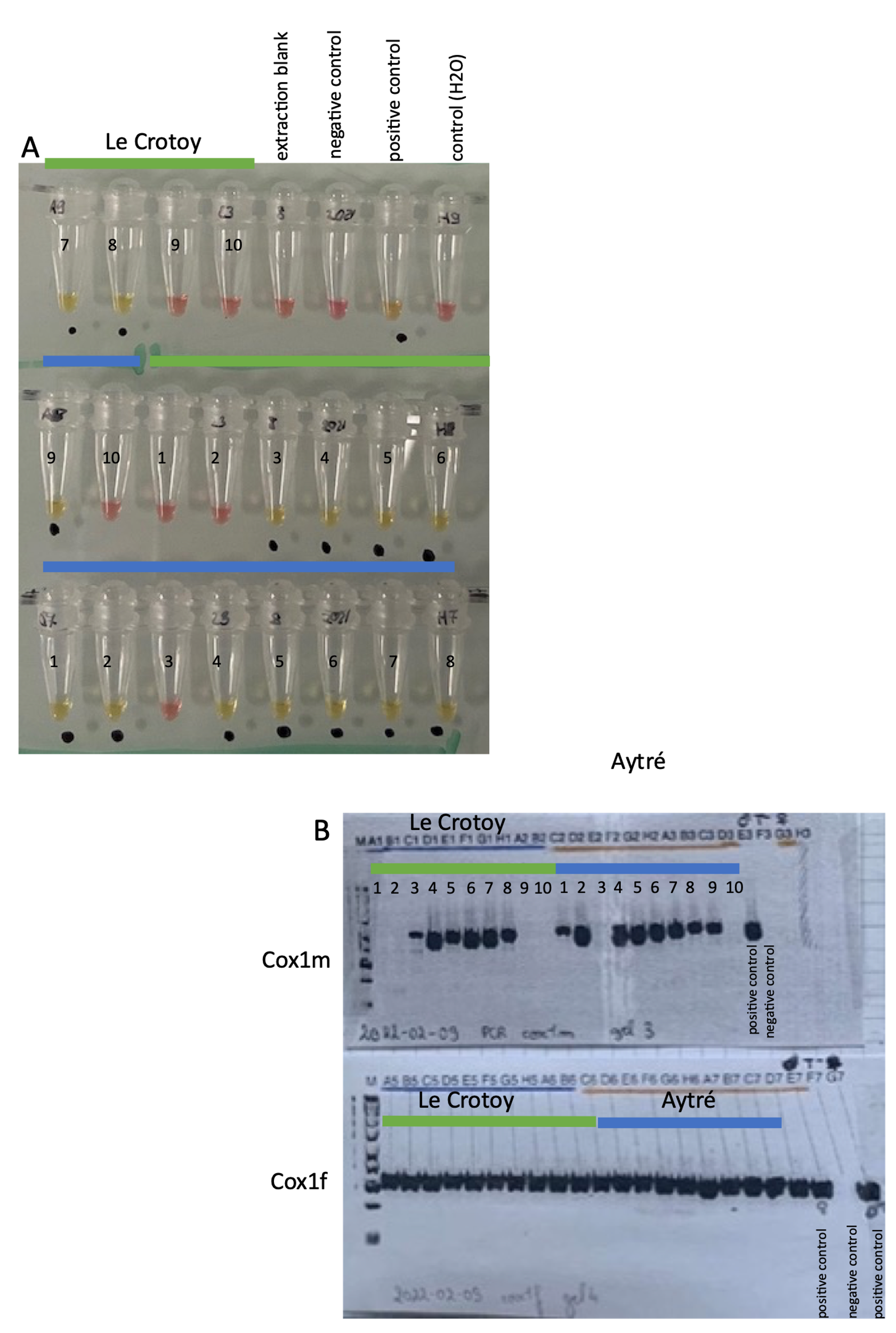

Supplement: Supplementary file 1 — Figure S1 [file ECE3-13-e10320-s001.docx]
